# Benchmarking outcomes for distal pancreatectomy: critical evaluation of four multicenter studies

**DOI:** 10.1007/s00423-023-02972-8

**Published:** 2023-06-29

**Authors:** P. C. Müller, J. M. A. Toti, C. Guidetti, C. Kuemmerli, M. Bolli, A. T. Billeter, B. P. Müller

**Affiliations:** 1 Department of Surgery, Clarunis, – University Centre for Gastrointestinal and Hepatopancreatobiliary Diseases, 4002 Basel, Switzerland; 2 Swiss HPB and Transplantation Center, Department of Surgery and Transplantation, University Hospital Zurich, Zurich, Switzerland; 3 Hepato-Pancreato-Biliary Surgery and Liver Transplantation Unit, University of Modena and Reggio Emilia, Modena, Italy

**Keywords:** Pancreatic surgery, Distal pancreatectomy, Robotic surgery, Outcome research, Complications

## Abstract

**Background:**

Benchmarking is a validated tool for outcome assessment and international comparison of best achievable surgical outcomes. The methodology is increasingly applied in pancreatic surgery and the aim of the review was to critically compare available benchmark studies evaluating distal pancreatectomy (DP).

**Methods:**

A literature search of English articles reporting on benchmarking DP was conducted of the electronic databases MEDLINE and Web of Science (until April 2023). Studies on open (ODP), laparoscopic (LDP), and robotic DP (RDP) were included.

**Results:**

Four retrospective multicenter studies were included. Studies reported on outcomes of minimally invasive DP only (*n* = 2), ODP and LDP (*n* = 1), and RDP only (*n* = 1). Either the Achievable Benchmark of Care™ method or the 75th percentile from the median was selected to define benchmark cutoffs. Robust and reproducible benchmark values were provided by the four studies for intra- and postoperative short-term outcomes.

**Conclusion:**

Benchmarking DP is a valuable tool for obtaining internationally accepted reference outcomes for open and minimally invasive DP approaches with only minor variances in four international cohorts. Benchmark cutoffs allow for outcome comparisons between institutions, surgeons, and to monitor the introduction of novel minimally invasive DP techniques.

## Introduction

In recent years technical advancement of laparoscopic and robotic-assisted surgery has led to less invasive procedures in the field of pancreatic surgery. Distal pancreatectomy (DP) is frequently performed by a minimally invasive approach (MIDP). Outcomes of MIDP have been assessed in randomized controlled trials (RCT), highlighting advantages such as reduced blood loss and faster recovery after surgery [1–5].

Currently, a shift from laparoscopic (LDP) to robotic DP (RDP) can be observed in international high-volume institutions, while the open approach still remains a valuable option, especially in advanced tumors infiltrating surrounding tissue.

There is an increasing need for validated tools for outcome assessment and international comparison. In this context benchmarking is a well-established technique to define expected outcomes of a certain process, allowing internal quality assessments, unbiased identification of performance gaps between centers, and finally comparison of subgroups of different preoperative risk populations. Its application in pancreatic surgery is rapidly diffusing in recent years [6, 7].

Mainly two different methods have been described to define benchmark outcomes for surgical procedures [8, 9]: The first method is a quality enhancement process from the realm of manufacturing and economy [10], which uses best performance in a given field as reference point for others to improve and aims to establish benchmark cutoffs as the 75th percentile obtained in a selected low-risk population called the “*benchmark patient cohort*.”

The second method is called the “Achievable Benchmark of Care™” (ABC™), developed in the early 2000s in the USA specifically to benchmark care process indicators with the aim of being able to assess the level of quality delivered by a certain care process and compare it with other process providers, e.g., other institutions or healthcare systems. ABC™ defines benchmark values as the best achievable outcome calculated as the proportion between the studied outcome and the number of cases performed by top performers arbitrarily calculated as the best 10% of the total population, taking into account the number of cases per center using an adjusted performance fraction. Recently, four different studies on defining benchmark outcomes for DP have been published [11–14]. The study population, design, and methodology of the four publications are different; thus, the present article aims to summarize and critically appraise their outcomes and assess their clinical implications.

## Methods

A literature search including the keywords “distal pancreatectomy” AND “benchmarking” was conducted of the electronic databases MEDLINE (via PubMed) and Web of Science to identify relevant studies published until April 2023. The following inclusion criteria were applied:adult patients (> 18 years) undergoing DP with an open, laparoscopic or robotic approachwith information on benchmark methodology and benchmark outcomespublished in English

Eligibility assessment and data extraction was performed independently in an unblinded manner by two reviewers (JMAT and CG). To avoid errors in data extraction, a double data-entry method was applied. Two authors (PCM and CG) compared the data and discussed discrepancies to achieve consensus. For each study we evaluated the study design, the methodology for defining the benchmark cut-offs, and finally the benchmark values of intraoperative and postoperative parameters itself.

## Results

### Study design

The study methodology is summarized in Table 1. All four studies utilized a multicenter retrospective study design. Two of the studies included 31 European centers participating at the European Consortium on Minimally Invasive Pancreatic Surgery (E-MIPS) [13, 14], one study 21 French high-volume centers [11], and the last one enrolled 16 international high-volume centers from three continents [12]. The center selection for all studies was based on case volume, and for one, an already established robotic pancreatic surgery program with a prospective database was a mandatory selection criterion [12]. For two studies the annual case load cutoff was set at 15 DPs/year [13, 14], for one at 10 RDPs/year, and for one a minimum of 20 pancreatic resections per year were required.

**Table 1:** Characteristics of the four included benchmark studies

	Giani et al	Van Ramshorst et al	Durin et al	Müller et al
Study design	Retrospective	Retrospective	Retrospective	Retrospective
Required DP case load/year	15	15	Not reported^†^	10^‡^
Center origin	Europe	Europe	France	Europe, USA, Asia
Number of centers	31	31	20	16
Study period	2003–2019	2006–2019	2014–2018	First RDP–2020
Inclusion criteria				
Age (years)	> 18	> 18	> 18	> 18
Indication for surgery	Benign/malignant	Benign/malignant	Benign/malignant	Benign/malignant
Type of surgery	MIDP with splenectomy	MIDP without splenectomy	Open and MIDP + / − splenectomy	Robotic DP + / − splenectomy
Exclusion criteria				
Borderline or locally advanced, metastatic lesions	ND	+	+	+
Learning curve	ND	ND	ND	+
Unplanned splenectomy	+	ND	ND	ND
Benchmarks				
Benchmark analysis method	ABC™	ABC™, low-risk subgroup	Low-risk subgroup	Low-risk subgroup
Study sample size (*n*)	1,819	1,230	1,188	755
Low risk benchmark cohort (%)	-	62^*^	63	46

† 20 pancreatectomies per center not only DP

‡ 10 RPD/ year and 50 pancreatectomies/ year

*% of low-risk population by determining benchmarks with Rössler et al. method

*MIDP*, minimally invasive distal pancreatectomy, *ABC™* Achievable Benchmark of Care; *ND*, not defined

### Patient selection

All four studies included patients with both benign and malignant lesions aged > 18 years. Only the French study included patients undergoing both MIDP and open DP (*n* = 749) [11]. The three other studies included only patients undergoing MIDP. While the two studies from the E-MIPS registry included both LDP and RDP [13, 14], the international multicenter study focused on RDP only.

Of the two E-MIPS studies, one included only patients who underwent MIDP with splenectomy (*n* = 1595) [13], while the other one only included patients who underwent MIDP with splenic preservation (SPDP) [14]. The two other studies included both, patients with and without splenectomy [11, 12].

Only one study accounted for the learning curve of MIDP and excluded the first 10 RDP per center to minimize the effects of the learning curve [12]. Exclusion criteria were homogeneously among the studies, patients with extensive resections according to ISGPS, borderline or locally advanced lesion), and emergency operations were excluded [15].

### Benchmark methodology

Among the four studies, two different methods were used to define the benchmarks. Müller et al. and Durin et al. used the traditional method firstly described by Rössler et al. [16] using the 75th percentile from the median of each center as the benchmark cutoff. The ABC™ method was used by Giani et al., whereas the study by Van Ramshorst et al. used both methods and compared them to each other. In defining the low-risk patient population for the benchmark analysis using the 75th percentile, Müller et al. and Durin et al. used the same criteria as those used in previous publications that defined pancreatic surgery benchmarks [8]. The criteria used by van Ramshorst et al. differed only in the body mass index cut-off (> 35 kg/m^2^ vs. > 40 kg/m^2^).

### Benchmark values

Benchmark cutoffs using the 75th percentile method are summarized in Table 2. Regarding intraoperative outcomes, cutoffs for operative times ranged from 232 to 300 min and blood loss from 150 to 195 ml. The conversion rate for pure RDP was low with 3 to 8%, while in the mixed cohort of LDP and RDP, it was between 6 and 20%. The failure rate to preserve the spleen was reported in one study with similar values between RDP (27%) and LDP (30%).

**Table 2:** Comparison of 75th percentile benchmark values

Benchmark cutoffs	Giani et al.^‡^	Van Ramshorst et al		Durin et al	Müller et al
	(*n* = 1595)	LSPDP (*n* = 602)	RSPDP (*n* = 162)	(*n* = 749)	(*n* = 345)
Minimally invasive cases (%) Laparoscopic (%) Robotic (%)	100 82 18	100 100 0	100 0 100	47 - -	100 0 100
Operation time (min)	275	254	263	232	300
Estimated blood loss (ml)	-	150	195	-	150
Conversion to open (%)	19.2	5.8	8.2	20	3
Failure to preserve spleen (%)	-	29.9	27.3	-	-
Complications at 3 months (%)	69.1	67.2	55.7	-	58.3
Major complications (≥ 3a) (%)	25.8	20.4	14	27.3	26.7
CCI at 3 months	-	-	-	-	8.7
Pancreatic fistula (B and C) (%)	30.5	23.8	24.2	28.6	31.8
Delayed gastric emptying (%)	-	-	-	-	5
Postoperative hemorrhage (%)	-	-	-	2.8	6.6
ICU stay (days)	-	-	-	25%*	1
Length of stay (days)	10	8	8	13	7
Readmission rate (%)	17	20	15	17	24
90-day mortality (%)	2.3	0	0	0	0
Oncological outcomes for PDAC					
Number of resected LN (n)	-	-	-	17.1**	9
R0-resection (malignant only) (%)	-	-	-	75.9	83
1-year OS (%)	-	-	-	88.3	87.3
3-year OS (%)	-	-	-	66.7	80
1-year DFS (%)	-	-	-	69.5	78.1
3-year DFS (%)	-	-	-	-	66.7

*CCI*^*®*^ Comprehensive complication index; *ICU*, intensive care unit; *LSPDP*, laparoscopic splenic preserving distal pancreatectomy; *PDAC*, pancreatic ductal adenocarcinoma; *RSPDP*, robotic splenic preserving distal pancreatectomy; *MIDPS*, minimally invasive distal pancreatectomy with splenectomy

‡75^th^ percentile of ABC benchmark distribution

°% of case with postoperative ICU stay

** Mean

Looking at postoperative outcomes, cutoffs for the overall morbidity varied from 56 to 69%, and for major complications (defined as Clavien-Dindo grade III or higher) ranged from 20 to 27% with the exception for the RDP cohort with an exceptionally low 14% [14]. Evaluating the rates of clinically relevant (grade B/C) postoperative pancreatic fistula (POPF), there was only a slight variation from 24 to 32%. Other pancreas specific outcomes such as delayed gastric emptying (DGE) and postoperative hemorrhage (PPH) were reported in a minority of studies. Length of stay ranged from 7 to 13 days, while the 13 days were reported for the cohort with 53% open DP. Readmissions rates were between 15 and 24% and benchmarks for 90-day mortality varied from 0 to 2.3%, respectively.

Two studies reported on oncological outcomes for patients with PDAC with cutoffs for R0 rate of 83% and 76%, respectively.

Cutoffs for best achievable results obtained with the ABC™ method are summarized in Table 3.

**Table 3:** Comparison of benchmark values in unselected group method ABC™

Benchmark cutoffs	Giani et al	Van Ramshorst et al	
	(n = 1595)	LSPDP (n = 951)	RSPDP (n = 279)
Operation time (min)	160	150	207
Estimated blood loss (ml)	-	55	100
Conversion to open (%)	2.5	2.5	3.5
Failure to preserve the spleen (%)	-	0	1.7
Complications at 3 months (%)	30.4	27.3	34
Major complications (≥ 3a) (%)	8.4	5.1	3.3
Pancreatic fistula (B and C) (%)	8.3	3.6	7.1
Length of stay (days)	5	5	6
Readmission rate (%)	4.1	2.9	5.4
90-day mortality (%)	0	0	0

*ABC*^*™*^ Achievable Benchmark of Care; *LSPDP*, laparoscopic splenic preserving distal pancreatectomy; *RSPDP*, robotic splenic preserving distal pancreatectomy

The results differ significantly from the above-mentioned methodology. Operative time ranged from 150 to 208 min and intraoperative blood loss from 55 to 100 ml. Benchmarks for conversion rate were 2.5–3.5%. Failure to preserve the spleen was 0% in the laparoscopic group and 1.7% in the RDP group. Overall complications at 3 months, major complications, and POPF rate were between 27 and 34%, 3.3 and 8.4%, and 3.6 and 8.3%, respectively. Cutoffs for length of stay, readmission-, and 90-day mortality were 5 to 6 days, 2.9 to 5.4%, and 0%, respectively. No oncological outcome was evaluated with the ABC™ method.

## Discussion

The goal of setting benchmark values is to improve patient outcomes by challenging surgical teams to achieve excellence. Furthermore, having benchmark values from international centers of excellence provides unambiguous reference values that can be used to assess new techniques, to compare outcomes between different institutions, and finally to critically evaluate the individual performance of each surgeon.

In this review on benchmarking open and minimally invasive DP, the four included studies applied two very different methodologies to define benchmarks. Both benchmark methods assumed that in order to obtain reproducible values, they must be derived from a population that is selected as the “*benchmark population*.”

With the first method (75th percentile) the benchmark population is derived from preoperative patient characteristics as detailed by the Delphi consensus by Gero et al. [8] This low-risk population has an ideal perioperative risk profile and the outcomes are assumed to be the best achievable due to the beneficial patient characteristics [16, 17].

The second method (ABC™) consists of a more complex selection process to define the benchmark population. As a first step, a classification of the providers (individual institutions) producing the considered outcome is performed from top to bottom. From this ranking, which takes into account the number of patients per center, 10% of events are then derived, thus creating a super-selected population from which benchmark values are obtained [18, 19].

Looking at the methodology used to calculate benchmark cutoffs, the different methods identify two opposite tails of a similar outcome distribution. For the first method the benchmark of each variable is derived from the 75th percentile from the median of each center. In the ABC™ method the benchmark is obtained as the proportion between the frequency of the event and the number of cases in the denominator, which finally turns out to be only the top 10% of the total population. For continuous variables, on the other hand, it is arbitrarily set at the 10th percentile of the normal distribution. Simply put, the first method evaluates the outcomes derived from an ideal low-risk population by claiming that they are within benchmark cutoffs, if they remain within the best 75% for that outcome. The second method instead selects the top 10% of all performers among the enrolled institutions, suggesting that you have to aspire to these excellent results to achieve benchmark outcomes. Given the methodological differences, a direct comparison between the benchmarks calculated with the two different methods is not meaningful. Instead, with a good margin of approximation, we could consider the benchmarks calculated in the low-risk population and defined by the 75th percentile to be the minimum outcomes to qualify as a good care provider, whereas the ABC™ benchmarks are the ones to strive for in order to be considered in the top 10% of performers.

Before delving into the differences of the clinical outcomes, one has to keep in mind that three studies calculated benchmarks at the 75th percentile from different surgical populations [11, 12, 14]. Van Ramshorst et al. only considered MIDP with splenic preservation, Durin et al. included both open and MIDP cases irrespective of splenic preservation, and Müller et al. only considered RDP, again with and without splenectomy. Otherwise, Giani et al. not only analyzed their results with the ABC™ method but by also reported the outcomes for the 75th percentiles of this cohort.

With regard to the specific differences of the benchmark cutoffs, there was minimal variation for operating times with 232 min up to 300 min. The first value is derived from the study with open DP, an approach associated with faster operation time [11], while the longer operation time was observed in the robotic-only series [12]. As far as the conversion rate is concerned, two trends can be observed, the first one with a low conversion rate ranging from 3 to 8.2% in RDP [12, 14] and the second one with rates of 5.8 to 20% for the laparoscopic approach [11, 13]. The learning curve seems to have a significant influence on the conversion rate as demonstrated by a doubled conversion rate within the first 10 RDP versus later in the international benchmark study on the robotic approach (6% vs. 3%) [12, 20].

Similar to the benchmark cutoffs for conversion, overall morbidity showed a difference between RDP and LDP. Benchmark values for overall morbidity were 10% reduced for the robotic approach (58%) as compared to LDP (69%). For the study including open cases, overall morbidity was not calculated [11]. However, the aforementioned differences seem to be due to less minor complications (CD grade < 3) as the rate of major complications is similar in all studies (20–27%). The single most important complication contributing to major morbidity in DP is POPF. Again, the rate of clinically relevant POPF uniformly ranged from 24 to 32% among studies and surgical approaches. Importantly, other pancreas-specific complications such as DGE (5%) and PPH (3–7%) were only evaluated in a minority of studies and need further research [11, 12].

Benchmark cutoffs for length of stay and readmission rate on the other hand were calculated in each study. Unsurprisingly, the series including open cases had the highest benchmark cutoff for hospital stay with 13 days, while hospital stay for MIDP varied between 8 and 10 days. Readmission rate varied between 15 and 24% and the 90-day mortality was generally low (max. 2.3%). Oncologic benchmark cutoffs for patients with PDAC were limited on values for R0 resection rate (> 76%) and number of lymph nodes harvested (> 9); those values need to be better defined in adequate patient cohorts.

Clinical outcomes of DP with or without splenectomy seem not to justify for separate benchmark outcomes of these two procedures. In a propensity score-matched UK-wide multi-center study, Moekotte et al. found no differences in perioperative outcomes comparing patients undergoing MIDP with and without splenectomy [21]. These results were furthermore confirmed in the international RDP analysis, therefore advocating for uniform DP benchmarks irrespective of splenectomy [12].

The excellent benchmarks assessed by the ABC™ approach should be carefully interpreted considering the methodology used to define them: selecting the top providers for each outcome and thereof only 10% of the population with best possible outcomes. This makes us question whether the population to define the benchmarks is ultimately truly representative and thus reproducible. Comparing the ABC™ benchmarks and 75th percentile cutoffs, we found the greatest differences (> 10%) between the benchmarks for overall complications, major complications, POPF, and readmission rate. This allows us to assume that in these areas is most room for improvement.

Recently, alternative concepts have been introduced to assess and compare multidimensional ideal outcomes in pancreatic surgery [5, 22, 23]. Textbook outcome (TO) is an expert consensus-based composite endpoint defined by the absence of all of the following parameters: POPF, bile leak, PPH, > 2 CD grade complications, readmission, and in-hospital mortality. In a nationwide Dutch analysis, TO was achieved in 67% for DP and 58% for pancreatoduodenectomy. Compared to the classical benchmarking concept, TO does not include intraoperative or oncologic parameters, thus representing an easily applicable composite endpoint to compare outcomes between different institutions. While TO generally does not take into account the risk profile of the included patients, van Roessel et al. found only female sex and absence of neoadjuvant therapy to be associated with a better TO rate in multivariate analysis [22].

With the ever-growing high-level evidence in pancreatic surgery, another popular concept is evidence mapping. This approach summarizes randomized controlled trials and presents outcomes as a living meta-analysis (https://emps.evidencemap.surgery/). While the outcomes in the living meta-analysis are derived from less homogenic patient cohorts than in classical benchmarking, these values may very well represent best achievable results in “real-world” scenarios. Furthermore, this approach may be especially valuable to provide benchmarks for often used (primary) outcomes in RCTs on pancreatic surgery such as POPF or DGE [5].

As a limitation of all benchmark studies, the inclusion of participants by center was not evenly distributed. Therefore, the benchmark values may be biased by volume-outcome relationships. Furthermore, only one study accounted for the learning curve of MIDP, arbitrary excluding the first ten performed cases from each center [12]. While there is no internationally accepted assessment or definition of “*the learning curve*” in pancreatic surgery, a recent systematic review found a learning period of 15 cases for both LDP and RDP. More importantly, the study showed that in a first phase (*competency*), mainly intraoperative parameters such as operative time, conversion rate, and blood loss improve, while in a later stage (*proficiency*/*mastery*) postoperative complications show a more pronounced improvement [20]. As a consequence, benchmarking of novel surgical approaches such as robotic surgery should include a rigorous learning curve assessment and elimination of cases performed during the learning curve.

In conclusion, benchmarking has shown to be a robust and reproducible tool for obtaining internationally accepted reference values for the different DP approaches in four international cohorts with only minor variances. Compared to LDP, benchmark outcomes for RDP show a decreased conversion rate and less overall complications. The presented benchmark cutoffs for DP allow comparisons between institutions, individual surgeons, and to assess the safety of new minimally invasive DP techniques such as RDP.

## Data Availability

Data sharing is not applicable to this article as no new data were created or analyzed in this study.

## Abbreviations

ABC^TM^: Achievable Benchmark of Care™
CCI®: Comprehensive complication index
CD: Clavien-Dindo classification
DGE: Delayed gastric emptying
DP: Distal pancreatectomy
ICU: Intensive care unit
ISGPS: International Study Group of Pancreatic Surgery
LDP: Laparoscopic distal pancreatectomy
MIDP: Minimally invasive distal pancreatectomy
POPF: Postoperative pancreatic fistula
PPH: Postpancreatectomy hemorrhage
RCT: Randomized controlled trials
RDP: Robotic distal pancreatectomy
SPDP: Splenic preserving distal pancreatectomy
TO: Textbook outcome
